# Polycaprolactone Nanofibers Functionalized by Fibronectin/Gentamicin and Implanted Silver for Enhanced Antibacterial Properties, Cell Adhesion, and Proliferation

**DOI:** 10.3390/polym16020261

**Published:** 2024-01-17

**Authors:** Elizaveta S. Permyakova, Anastasiya O. Solovieva, Natalia Sitnikova, Philipp V. Kiryukhantsev-Korneev, Magzhan K. Kutzhanov, Alexander N. Sheveyko, Sergey G. Ignatov, Pavel V. Slukin, Dmitry V. Shtansky, Anton M. Manakhov

**Affiliations:** 1 Research Laboratory “Inorganic Nanomaterials”, National University of Science and Technology “MISIS”, Moscow 119049, Russia; permyakova.elizaveta@gmail.com (E.S.P.); kiruhancev-korneev@yandex.ru (P.V.K.-K.); makonyo95year@gmail.com (M.K.K.); sheveyko@mail.ru (A.N.S.); ignatov.sergei@gmail.com (S.G.I.); xopgi@yandex.ru (P.V.S.); shtansky@shs.misis.ru (D.V.S.); 2 Research Institute of Clinical and Experimental Lymphology—Branch of the ICG SB RAS, 2 Timakova St., Novosibirsk 630060, Russia; solovey_ao@mail.ru (A.O.S.); sitnikovanat9@gmail.com (N.S.); 3 State Research Center for Applied Microbiology and Biotechnology, Obolensk 142279, Russia; 4 Lomonosov Moscow State University, GSP-1, 1 Leninskiye Gory, Moscow 119991, Russia

**Keywords:** XPS, PCL nanofibers, fibronectin, Immobilization, surface functionalization, plasma, silver

## Abstract

Novel nanomaterials used for wound healing should have many beneficial properties, including high biological and antibacterial activity. Immobilization of proteins can stimulate cell migration and viability, and implanted Ag ions provide an antimicrobial effect. However, the ion implantation method, often used to introduce a bactericidal element into the surface, can lead to the degradation of vital proteins. To analyze the surface structure of nanofibers coated with a layer of plasma COOH polymer, fibronectin/gentamicin, and implanted with Ag ions, a new X-ray photoelectron spectroscopy (XPS) fitting method is used for the first time, allowing for a quantitative assessment of surface biomolecules. The results demonstrated noticeable changes in the composition of fibronectin- and gentamicin-modified nanofibers upon the introduction of Ag ions. Approximately 60% of the surface chemistry has changed, mainly due to an increase in hydrocarbon content and the introduction of up to 0.3 at.% Ag. Despite the significant degradation of fibronectin molecules, the biological activity of Ag-implanted nanofibers remained high, which is explained by the positive effect of Ag ions inducing the generation of reactive oxygen species. The PCL nanofibers with immobilized gentamicin and implanted silver ions exhibited very significant antipathogen activity to a wide range of Gram-positive and Gram-negative strains. Thus, the results of this work not only make a significant contribution to the development of new hybrid fiber materials for wound dressings but also demonstrate the capabilities of a new XPS fitting methodology for quantitative analysis of surface-related proteins and antibiotics.

## 1. Introduction

Wound healing is a complex process involving the stages of inflammation, re-epithelialization, angiogenesis, granular tissue formation, wound closure, and normal tissue formation. Materials intended for wound healing must have an extracellular matrix-like structure and a set of properties, such as biocompatibility, ability to stimulate angiogenesis, and antibacterial activity, the violations of which can lead to ineffective healing and/or rejection of skin grafts [1,2]. The extracellular matrix (ECM) plays a major role in wound healing by regulating cell migration, proliferation, and angiogenesis [3]. Therefore, bioengineered skin substitute scaffolds for skin regeneration are usually designed by mimicking the properties of native ECM.

The scientific field of developing tissues for wound healing, called tissue engineering, is rapidly developing, attracting increasing interest from the scientific community. Tissue-engineered structures are often created using natural polymers such as collagen, chitosan, and others. Collagen nanofibers have a significant effect on promoting wound healing [4,5]. Spinning biodegradable polymer nanofibers is the most promising method, allowing the production of large-area nanofiber films with controlled morphology with a relatively simple process [6]. Nanofibers produced from natural polymers are biocompatible, but producing stable and uniform nanofibers is still challenging. In addition, the use of natural polymers increases the cost of fabrics due to the high cost of the polymers themselves, which prevents the scaling up of the process. For example, collagen costs about $45,000 USD per ton, which is about ten times the price of synthetic biodegradable polymers [7]. In addition, many natural polymers easily decompose in an aqueous environment, which opens new possibilities for bioactive nanofibers from synthetic polymers.

Synthetic biodegradable materials that provide the required concentrations and release rates of growth factors and antimicrobial chemicals are being actively developed. However, untreated synthetic polymer nanofibers exhibit poor cell adhesion and slow wound healing rates, so they are doped or subjected to surface modification. There are three most popular methods for modifying nanofibers: (i) co-spinning of biopolymers with nanofibers, (ii) gas discharge plasma treatment with the addition of growth factors, and (iii) plasma polymerization. For example, wound healing has been improved by attaching growth factors to plasma-treated polycaprolactone (PCL) and polylactide (PLA) nanofibers [8,9,10]. Energy-efficient and stable plasma polymerization is the most promising method for processing nanofibers. Plasma-coated PCL nanofibers with immobilized platelet-rich plasma (PRP) have been shown to be useful for the treatment of diabetic wounds [11,12,13].

Targeted tuning of the surface topography, microstructure, and chemical composition of synthetic polymer nanofibers is important in the development of novel nanomaterials for biological applications [4,14]. Recent results have shown that immobilization of viable biomolecules can enhance the bioactivity of polymers [15]. Improved surface bioactivity may be due to the immobilization of certain proteins, such as fibronectin and apolipoprotein [16,17]. Fibronectin, a vital protein found in the ECM, plays a crucial role in cell adhesion and migration, promoting soft tissue regeneration [18]. Thus, the attachment of proteins can significantly improve the efficiency of tissue therapy.

The structure and density of immobilized proteins have a major influence on the therapeutic efficacy of scaffolds used as cell grafts. Vital proteins such as enzymes, growth factors, and extracellular matrix components play important roles in various biological processes, including cell signaling, tissue regeneration, and immune responses. Therefore, complete or partial degradation of these proteins under high-energy ion bombardment accompanied by thermal effects can significantly affect their functionality and biological activity. Even polymers such as polycaprolactone (PLA) are susceptible to significant structural damage under relatively mild ion bombardment, as demonstrated by X-ray photoelectron spectroscopy (XPS) analysis [19]. Therefore, it is important to control the surface structure at each stage of the modification process. It is important to ensure a strong bond of proteins during immobilization in order to avoid their accelerated degradation. For example, covalent immobilization of fibronectin provides improved protection against proteolysis compared to non-covalent immobilization [15]. This can be explained by the fact that the ECM in chronic wounds is rapidly degraded due to the presence of a biological microenvironment with a high concentration of reactive oxygen species (ROS). These enzymes are responsible for degrading and changing the protein structure, so prolonged release is preferable. Thus, covalently immobilized fibronectin can serve as an important structural component to facilitate cell regeneration and prolonged protein release. The presence of weak bonds, on the contrary, promotes the rapid release and delivery of biologically active substances, which can lead to their accelerated degradation.

Another approach to modifying nano/submicron fibers is magnetron sputtering and/or ion implantation of various metals to achieve improved characteristics. The incorporation of metal nanoparticles (NPs) or their oxides (for example, Au/AuO [20], Ag/Ag_2_O, Cu/CuO [21], Mg/MgO [22], etc.) into the structure of a biodegradable fiber mat has a positive effect on growth and cell proliferation [23,24,25]. At the same time, the therapeutic efficiency of polymer scaffolds depends on the presence of antibacterial properties that prevent or slow down inflammation. The most commonly used are Ag nanoparticles (AgNPs), the bactericidal effect of which is explained by two main factors: (i) the accumulation of AgNPs in mitochondria, leading to their dysfunction, and (ii) the destruction of the DNA structure, which contributes to the effective suppression of bacterial multiplication [26]. It is of interest to combine the two approaches, i.e., protein immobilization and bactericide ion implantation, to achieve a synergistic effect; however, it should be taken into account that ion implantation can lead to protein degradation.

Currently, information on the chemical bonds between proteins and the surface, as well as on the density of biomolecules, is quite limited. The main attention in the study of protein-surface interactions was paid to immobilized fibronectin [15,16,17]. Methods for detecting fibronectin are complex and require the use of antibody staining. In most studies, fibronectin immobilization was assessed qualitatively by XPS by increasing the nitrogen content or changing the carbon environment by “standard” fitting the C1s signal, introducing basic carbon environments [20]. Quantitative approaches for determining peptides and proteins by XPS based on such “standard” simple C1s curve fitting were studied before [16,18,19], but it is not enough to obtain reliable information on the density of biomolecules. The XPS C1s curve fitting technique for studying the degradation of biomolecules is complex and requires careful analysis of the peak shape and assessment of the background contribution. Recently, we developed a new approach and used it for polymers and biomolecules. It was never applied for investigation of the fibronectin immobilization and degradation. However, such an analysis can provide useful information about the effect of degradation on the biomolecule chemical composition and serve as a basis for the development of further approaches to improve their stability.

In the present work, PCL nanofibers coated with a plasma layer rich in COOH groups were fabricated. Fibronectin and gentamicin were attached to the nanofiber surface. Additionally, PCL nanofibers with protein and antibiotic attached were implanted with Ag ions. The sample chemical structure was studied based on a new XPS approach to fitting the C1s curve after each modification step. Finally, the biological characteristics of the resulting nanofibers were evaluated.

## 2. Materials and Methods

### 2.1. Electrospinning of PCL Nanofibers

Nanofibers were obtained by electrospinning a 25% PCL solution. To dissolve granulated PCL, 99% acetic acid and 98% formic acid were used in a 2:1 weight ratio. All materials were purchased from Sigma Aldrich (Darmstadt, Germany). The samples were electrospun on a SuperES-2 machine from ESpin Nanotech (ESpin Nanotech, Kanpur, India) using a drum collector (700 rpm). The flow rate of the PCL solution was 0.13 µL/sec. Samples were collected on a polypropylene cloth located at a distance of 18 cm from the nozzle. The electrospinning voltage was kept constant at 35 kV. Untreated, as-prepared PCL nanofibers are designated as PCL-ref. The PCL scaffold was 100 µm thick. Additional details on the electrospinning method may be found elsewhere [27].

### 2.2. Plasma-Deposited Coating, Containing Carboxyl Group-(-COOH) and Ag Ion Implantation

The deposition of COOH polymer layers was carried out utilizing a UVN-2M vacuum unit equipped with rotary and oil-diffusion pumps. The plasma was activated using a radio frequency (RF) Cito 1310-ACNA-N37A-FF power supply unit (Comet, Flamatt, Switzerland). The power source was connected to an RFPG-128 disk generator (Beams & Plasmas, Moscow, Russia) installed in a vacuum chamber. The duty cycle was 5%, and the RF power was maintained at 500 W. The residual pressure in the reactor was less than 10^−3^ Pa. The following gases were supplied to the vacuum chamber: CO_2_ (99.995%), Ar (99.998%), and C_2_H_4_ (99.95%). Gas flows were controlled using a 647C Multi-Gas Controller (MKST, Newport, RI, USA). The argon (Ar), carbon dioxide (CO_2_), and ethylene (C_2_H_4_) flow rates were adjusted to 50, 16.2, and 6.2 sccm, respectively. The pressure in the vacuum chamber was monitored using two types of controllers: VMB-14 (Tokamak Company, Dubna, Russia) and D395-90-000 (Edwards Limited, West Sussex, UK). The RF electrode and the substrate were positioned at a distance of 8 cm from each other. The deposition time was 15 min, resulting in a plasma layer approximately 100 nm thick. Plasma-coated PCL nanofibers are referred to as PCL-COOH.

Silver ions were implanted using a MEVVA-type implanter located at a distance of 180 mm from the sample and operating at an acceleration voltage of 10 kV and a current of 10 mA for 2.5 and 5 min [28]. The samples after Ag ion implantation were denoted as PCL-COOH-FBn-Ag (with attached fibronectin) and PCL-COOH-GM-Ag (with immobilized gentamycin).

### 2.3. Characterization

The microstructures of PCL-ref, PCL-COOH, PCL-COOH-FBn, and PCL-COOH-FBn-Ag fibers were examined using a JSM-7600F Schottky field emission scanning electron microscope (JEOL Ltd., Tokyo, Japan) equipped with an energy-dispersive X-ray (EDX) detector operating at 15 kV. To eliminate the surface charge and protect the sample from the electron beam, a Pt layer approximately 40 nm thick was deposited using a Smart Coater (JEOL Ltd., Tokyo, Japan). The specific surface area, including the pore size distribution of sample PCL-ref, was measured using a BET analyzer (AMI-300Lite, Altamira Instruments, Cumming, GA, USA). The chemical and phase compositions were examined using energy-dispersive X-ray (EDS) spectroscopy (EDXS) using an 80-mm^2^ X-Max EDX detector (Oxford Instruments, Abingdon, UK). X-ray photoelectron spectroscopy (XPS) studies were performed using a PHI VersaProbe III spectrometer (ULVAC-PHI Inc., Chigasaki, Japan). The device was equipped with a single-color Al Kα X-ray source (hv = 1486.6 eV). The studies were carried out at a pass energy of 23.5 eV and an X-ray output of 50 W. The spectra were analyzed using the CasaXPS software (version 2.3.16) after removing the Shirley-type background. The examined region has a maximum lateral resolution of 0.7 mm. Calibration of the binding energy (BE) scale was achieved by adjusting the position of the hydrocarbon CH_x_ component to 285.0 eV. The BEs for all carbon and oxygen environments were obtained from literature sources [14,29,30]. Infrared spectra were recorded in the range of 4000–370 cm^−1^ using a Vertex 80V Fourier transform infrared (FTIR) spectrometer (Bruker, Ettlingen, Germany) in attenuated total reflectance mode (ATR-FTIR). Data was collected at a pressure of 250 Pa with a resolution of 4 cm^−1^ and 100 scans.

### 2.4. Immobilization of Fibronectin and Gentamycin

To covalently attach fibronectin/gentamicin to plasma-treated nanofibers, samples were immersed for 15 min in a 2 mg/mL solution of 1-ethyl-3-(3-dimethylaminopropyl) carbodiimide (EDC) (Sigma Aldrich, 98%) in water. Samples were thoroughly cleaned in PBS before immersion in fibronectin solution (1 mg/mL) for 60 min. After treatment, the samples were carefully rinsed with PBS. These samples were designated as PCL-COOH-FBn. Gentamicin immobilization was carried out similarly, with the only difference being that instead of a fibronectin solution, an aqueous gentamicin solution (2 mg/mL) was used. The sample was designated as PCL-COOH-GM.

### 2.5. Cell Tests

The human fibroblast cell line (MRC5) of 20 passage was purchased from the State Research Center of Virology and Biotechnology “VECTOR” (Novosibirsk, Russia). Cells were cultured in Dulbecco’s modified Eagle’s medium (DMEM, Sigma Aldrich, St. Louis, MO, USA) supplemented with 10% fetal bovine serum (Gibco, Waltham, MA, USA) under standard culture conditions (humidified atmosphere, 5% CO_2_ and 95% air, at 37 °C).

Nanofiber samples PCL-ref, PCL-COOH, PCL-COOH-FBn, PCL-COOH-FBn-Ag2.5 PCL-COOH-FBn-Ag5 (Ag2.5 and Ag5 denote ion implantation time) were cut into round-shaped pieces with 0.5 cm diameter and used in the experiments. Cells were seeded at a concentration of 10 × 10^3^ cells per piece of scaffold. After 24 h, scaffolds were fixed with 4% % paraformaldehyde solution, and cells were stained with Alexa Fluor 532 phalloidin (Thermo Fisher Scientific, Waltham, MA, USA) after the cell membrane permeabilization by 0.1% and Triton X-100 and Hoechst 33,342 (Thermo Fisher Scientific, Waltham, MA, USA). Cells area was assessed by measuring the area phalloidin staining of cellular actin filaments, and cell counting was performed using Hoechst 33,342 cell nuclei staining. Cells were examined by fluorescence microscopy (Zeiss, Axio observer Z1, Oberkochen, Germany). The acquired images were processed using ZEN blue software version blue edition 04/2021 (Zeiss, Oberkochen, Germany). Experimental data was analyzed using Statistica 8.0 software using the nonparametric Mann–Whitney U test. Results are presented as mean ± standard deviation with statistical significance of *p* < 0.05.

### 2.6. Antipathogen Tests

The samples of unmodified and modified PCL nanofibers were tested against different pathogenic clinical strains, including Gram-negative bacteria (*Escherichia coli* ATCC25922, *Escherichia coli* U20, *Escherichia coli* U4, *Escherichia coli* K261, *Klebsiella pneumoniae* 67565/23, *Pseudomonas aeruginosa* 3945/23, *Proteus mirabilis* 3223/23), *Gram-positive bacteria* (*Staphylococcus aureus* BAA1707 (MW2), *Staphylococcus aureus* 11, *Staphylococcus aureus* 10708/23, *Enterococcus faecium* Ya253, *Enterococcus faecium* i237), and fungi (*Candida auris* KA10). The 0.1 mL of fresh suspension of tested microorganisms (0.5 McFarland standard, 10^6^ colony forming units (CFU)) in sterile physiological solution (NaCl, 9 g/L) was placed on the surface of Mueller-Hinton agar (HiMedia, Kennett Square, PA, USA) in the Petri dish. Then, the tested nanofibrous mats (size of 25 × 25 mm^2^) were placed with their coated side on the agar surface. The plates were incubated at 37 °C for 24 h and examined. The degree of antibacterial activity was evaluated by the diameter of the bacterial inhibition zone around the sample. The experiments were performed in triplicate, and statistical calculations were performed using the data analysis tool of Microsoft Excel 2010 (Microsoft Corp., Redmond, WA, USA)

## 3. Results

### 3.1. Chemistry and Morphology of Composite Nanofibers

**Figure 1:**
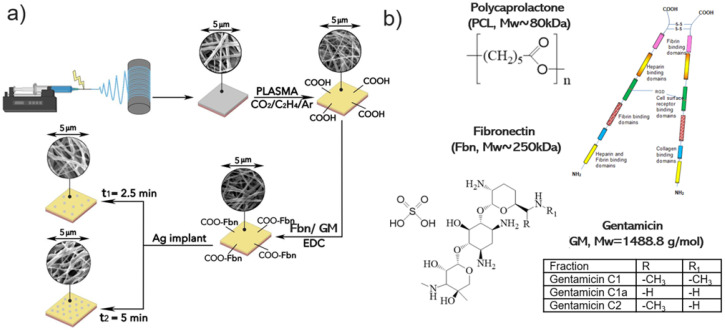
Schematics of nanofiber fabrication and their subsequent modification (**a**) and chemical structure of polymer and immobilized compounds (**b**).

**Figure 2:**
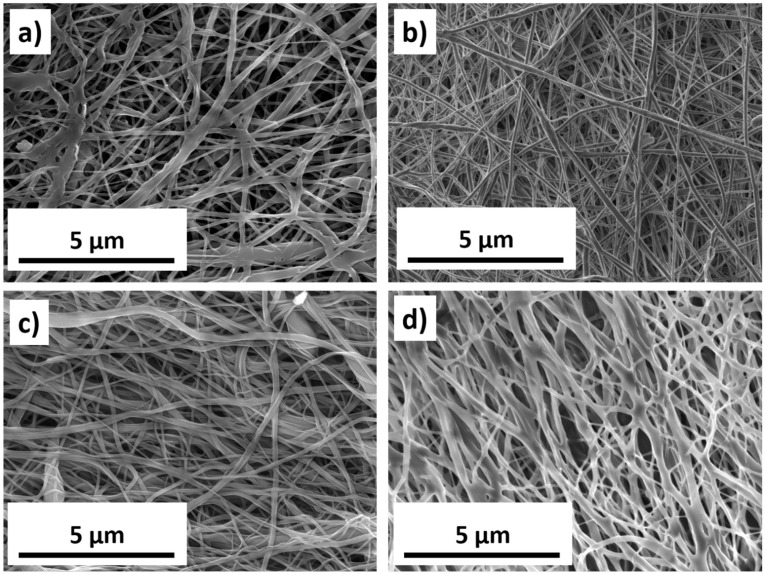
SEM micrographs of PCL-ref (**a**), PCL-COOH (**b**), PCL-COOH-FBn (**c**), and PCL-COOH-FBn-Ag2.5 (**d**) samples.

Schematics of nanofiber fabrication and their subsequent modification are presented in Figure 1. Figure 2 shows SEM micrographs of samples PCL-ref, PCL-COOH, PCL-COOH-FBn, and PCL-COOH-FBn-Ag. Based on the measurement of 100 randomly selected fibers from each sample using ImageJ software (version 1.53), the average size of the PCL-ref nanofibers was determined to be 270 ± 50 nm. The surface area and pore volume of the PCL-ref material were determined by nitrogen adsorption–desorption measurements. The BET surface area of sample PCL-ref is 24.90 m^2^/g, the total pore volume is 0.056 cm^3^/g, and the pore size distribution has a maximum of 9.1 nm. Note that the nanofibers remain intact after subsequent modifications associated with COOH deposition, protein attachment, and Ag ion implantation (Figure 2b–d).

EDXS analysis shows that Ag is uniformly distributed over the PCL-Ag fiber surface, although large Ag NPs agglomerates are also detected. The results of determining the sample chemical compositions using the EDXS method are summarized in Table 1. The Ag content of PCL-COOH-FBn-Ag2.5 and PCL-COOH-FBn-Ag5 fibers is 0.2 and 0.3 at.%, respectively. In addition, EDXS analysis of the front and back (covered by the substrate) sides of the samples shows that in the PCL-COOH-FBn-Ag5 material, Ag is present on both sides. This indicates that Ag ions penetrated through the nanofibers.

A comprehensive chemical study of the sample surfaces was carried out by XPS, the results of which are shown in Table 2. The atomic percentages of all elements were established by analyzing the high-resolution spectra of each component. Changes in the chemical composition of all surfaces are clearly visible, but information that is more important was obtained from high-resolution spectra fitting and analysis.

**Figure 3:**
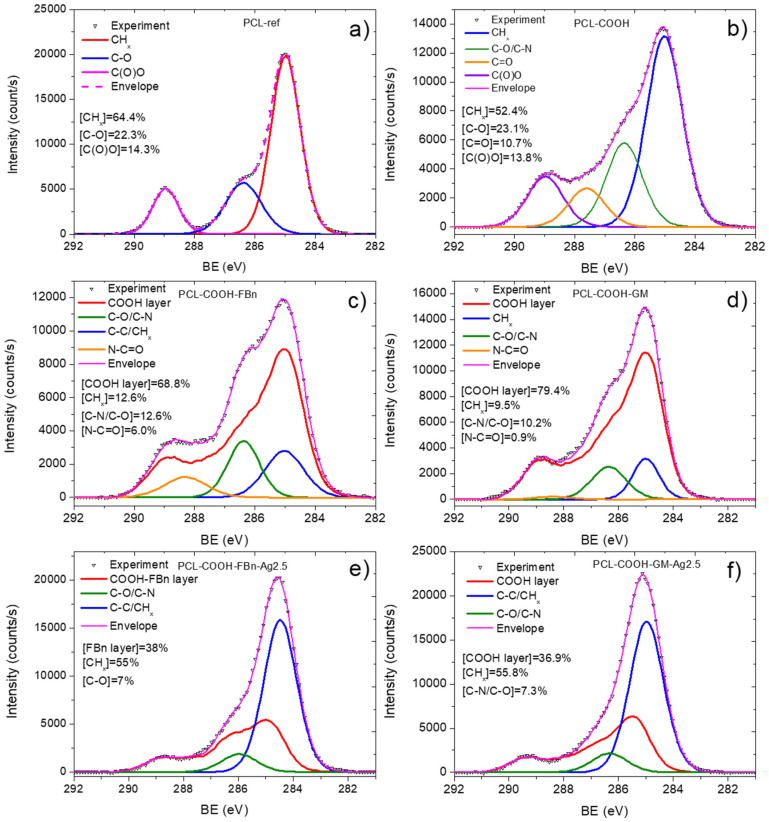
XPS C1 spectra of PCL-ref (**a**), PCL-COOH (**b**), PCL-COOH-FBn (**c**), PCL-COOH-GM (**d**), PCL-COOH-FBn-Ag2.5 (**e**), and PCL-COOH-GM-Ag2.5 (**f**) materials.

**Figure 4:**
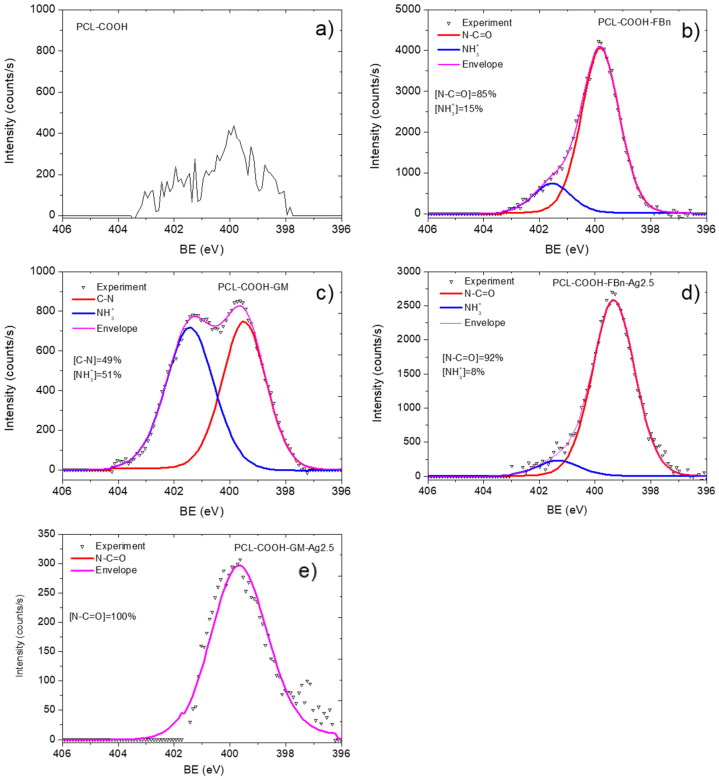
XPS N1 spectra of PCL-COOH (**a**), PCL-COOH-FBn (**b**), PCL-COOH-GM (**c**), PCL-COOH-FBn-Ag2.5 (**d**), and PCL-COOH-GM-Ag2.5 (**e**) materials.

The XPS C1s spectrum of sample PCL-ref (Figure 3a) is fitted by the sum of three components: hydrocarbons CH_x_ (BE = 285 eV), ether group C-O (BE = 286.4 eV), and ester group C(O)O (BE = 289.0 eV). The full width at half maximum (FWHM) of the C-O peak is 1.35 eV, and the FWHM of the CH_x_ and C(O)O components is 1.1 and 0.85 eV, respectively. The fitting of the XPS C1s spectrum of the PCL-COOH fibers was completely different. The spectrum was deconvoluted into four components: hydrocarbons CH_x_ (BE = 285.0 eV, used to calibrate the BE scale), carbon singly bonded to oxygen C-O (BE = 286.55 ± 0.05 eV), carbon doubly bonded to oxygen C=O/O-C-O (BE = 288.0 ± 0.05 eV), and C(O)O (BE = 289.1 ± 0.05 eV). Figure 3c displays the concentrations of all constituents. Traces of nitrogen were found in the PCL-COOH material (Figure 4a), which could be associated with surface contaminations.

Quite significant changes in the C1s and especially N1s spectra were observed after fibronectin immobilization. Nitrogen in the PCL-COOH-FBn material (Figure 4b) was attributed mainly to amide groups (N-C=O, BE = 400.0 eV, FWHM 1.7 eV) and protonated amines (NH_3_^+^, BE = 400.0 eV, FWHM = 1.7 eV). However, it is not possible to quantify the percentage of surface covered by proteins or therapeutic agents either from these simple observations or from standard C1s curve fitting. To solve this problem, a new method was used for the first time to quantify the content of biomolecules on the surface. This method was previously tested for complex mixed polymers [27].

Using CasaXPS software, the XPS C1s signal of the PCL-COOH-FBn fibers was approximated by introducing a new line that followed the waveform from the PCL-COOH counterpart. This method allows one to quantify the amount of plasma polymer surface. The C1s signal was fitted by a sum of four components: the plasma layer (PCL-COOH, BE = 285 eV), CH_x_ (BE = 285.0 eV, FWHM = 1.5 eV), C-N/C-O (BE = 286.4 eV, FWHM = 1.3 eV), and N-C=O (BE = 288.3 eV, FWHM = 1.7 eV).

The fitted of the XPS C1s spectrum of the PCL-COOH-FBn sample is shown in Figure 3c. According to the fitting results, more than 30% of the surface is covered with CH_x_, C-N/C-O, and N-C=O groups. The correlation between the atomic concentration of nitrogen and the N-C=O and C-N/C-O concentrations confirms the accuracy of our fitting. Indeed, if you simply count the number of nitrogen atoms in the C-N groups, you get [C-N/C-O] × [C]/100% = 8.4 at.%, which almost perfectly corresponds to the atomic concentration of nitrogen 8.0 at.% (Table 1).

The same approach was used to analyze the PCL-COOH-GM layer. However, in this case, the sum of the CH_x_, C-N/C-O, and N-C=O components was only 20% (Figure 3d). However, this is quite expected since the nitrogen concentration is not high (2.9 at.%). Indeed, for a correct comparison of the calculated C-N/C-O and nitrogen atomic concentrations, it is necessary to take into account that the structure of the gentamicin contains seven C-N and eight C-O bonds and, thus, when analyzing [C-N/C-O] concentration, it contains 7/15 atoms of C-N and 8/15 atoms of C-O. Taking this correction into account, we estimated that the concentration of C-N atoms is 7/15 × 10% × 69.4 at.%/100 = 3.2 at.%. This value correlates well with nitrogen contrition (Table 1). Also, the fitting of the N1s signal confirmed that the surface of the PCL-COOH-GM sample consists of C-N and NH_3_^+^ environments, i.e., amines and protonated amines, respectively. Therefore, our proposed methodology can correctly characterize the amount of gentamycin on the PCL-COOH-GM surface.

### 3.2. Analysis of Ag-Implanted Samples

Our XPS fitting methodology was also applied to analyze samples after Ag ion implantation. In this case, the XPS fitting analysis is more complex. Apparently, ion bombardment caused significant changes in the composition of PCL-COOH-GM and PCL-COOH-FBn materials. As can be seen in Table 1, the Ag concentration is very low. Despite the rather “soft” energy conditions of the implantation process, samples PCL-COOH-GM-Ag and PCL-COOH-FBn-Ag have a significantly lower nitrogen content, which, however, does not reflect the full picture of the surface chemical degradation. Fitting the C1s signals of samples PCL-COOH-FBn-Ag and PCL-COOH-GM-Ag shows a transformation of the surface structure mainly into graphite or hydrocarbon (Figure 3e,f). Using CasaXPS software, the XPS C1s signal of sample PCL-COOH-FBn-Ag was approximated by introducing a new curve that followed the waveform from the PCL-COOH-FBn material. The percentage of this component reflects the “preservation” of initial chemistry after Ag ion implantation. As follows from Figure 3e, the content of the “FBn layer” is 38%. Thus, it can be concluded that more than 60% of the surface chemistry was degraded, mainly towards hydrocarbons, since the CH_x_ component accounts for about 55%. The XPS N1s spectra of samples PCL-COOH-FBn-Ag and PCL-COOH-GM-Ag, depicted in Figure 3d and 3e, respectively, also indicate significant changes in the nitrogen environment. In both materials, almost all nitrogen atoms were attributed to amide groups.

**Figure 5:**
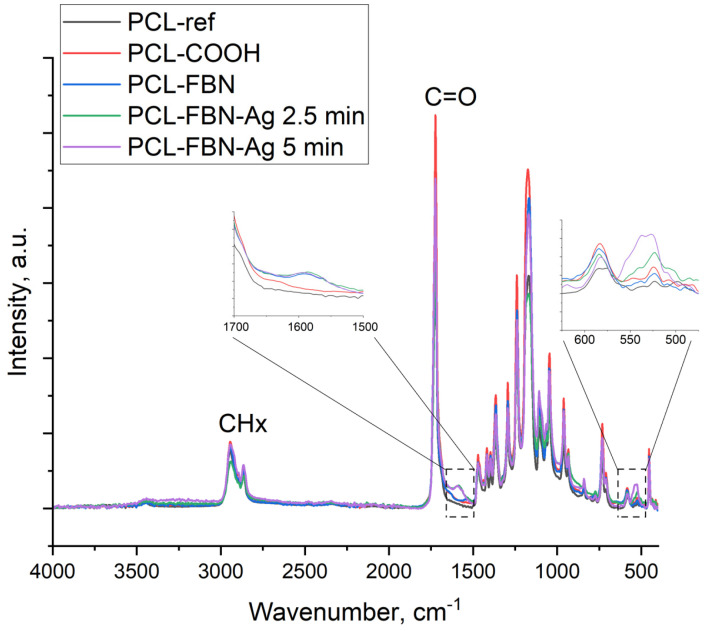
FTIR spectra of samples PCL-ref, PCL-COOH, PCL-COOH-FBN, PCL-COOH-FBN-Ag2.5, and PCL-COOH-FBN-Ag5. The insets show enlarged regions of the spectra, highlighted by dashed squares.

For additional surface characterization, FTIR spectroscopy was used. The obtained FTIR spectra are presented in Figure 5. All samples exhibited characteristic features inherent in the structure of polycaprolactone [25,26]: the peak at 1720 cm^−1^ is attributed to the carbonyl group of the ester group, peaks in the range of 3000–2800 cm^−1^ are assigned to the asymmetric and symmetrical C-H_2_ stretching, and maxima at 1300 and 1000 cm^−1^ are characteristic of CO stretching. Absorption bands at 1448 cm^−1^ (in-plane vibrations) and 988 cm^−1^ (out-of-plane vibrations) evidence the presence of C-O-H (carboxylic acid). There are also H-C-H bending vibrations observed at 1400 cm^−1^. Treatment of the PCL-ref sample in plasma did not lead to significant changes in its IR spectrum due to the similarity of the chemical composition of the plasma-deposited polymer with the structure of polycaprolactone [22]. Immobilization of fibronectin led to the formation of a peptide bond (characteristic peak at 1650 cm^−1^) [27]. Ag ion implantation led to the appearance of an additional peak located around 550 cm^−1^, which was attributed to Ag oxide lattice vibrations [28,29].

### 3.3. Cell Tests

The biological activity of biomaterials influences the behavior of cells in contact with the surface. The ECM is not only a tissue component but also constitutes the physical basis of cells. ECM transmits biochemical and biomechanical stimuli necessary for tissue morphogenesis and homeostasis. Due to their ability to modulate cell behavior, ECM proteins are of great scientific interest. The surface composition of a biomaterial determines key characteristics of the cellular response, including adhesion, migration, proliferation, and differentiation [30]. Silver exhibits antibacterial properties, so it is actively used as a bactericidal additive. Below, we describe the results of experiments to determine the effect of Ag ion bombardment on the functional activity of fibronectin immobilized on the surface of PCL nanofibers.

**Figure 6:**
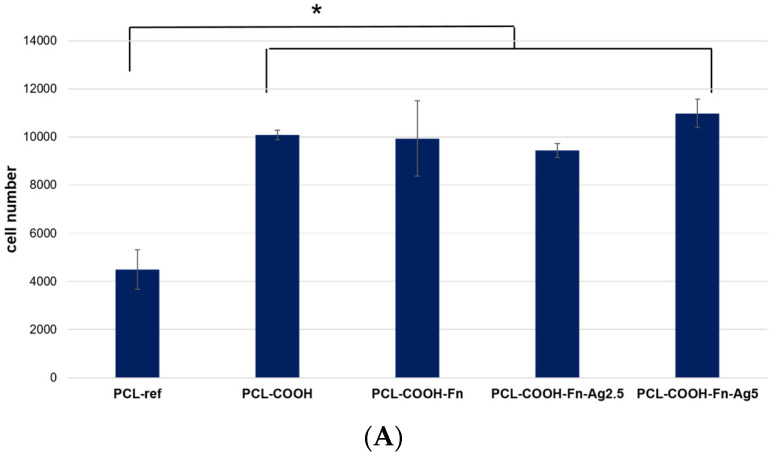

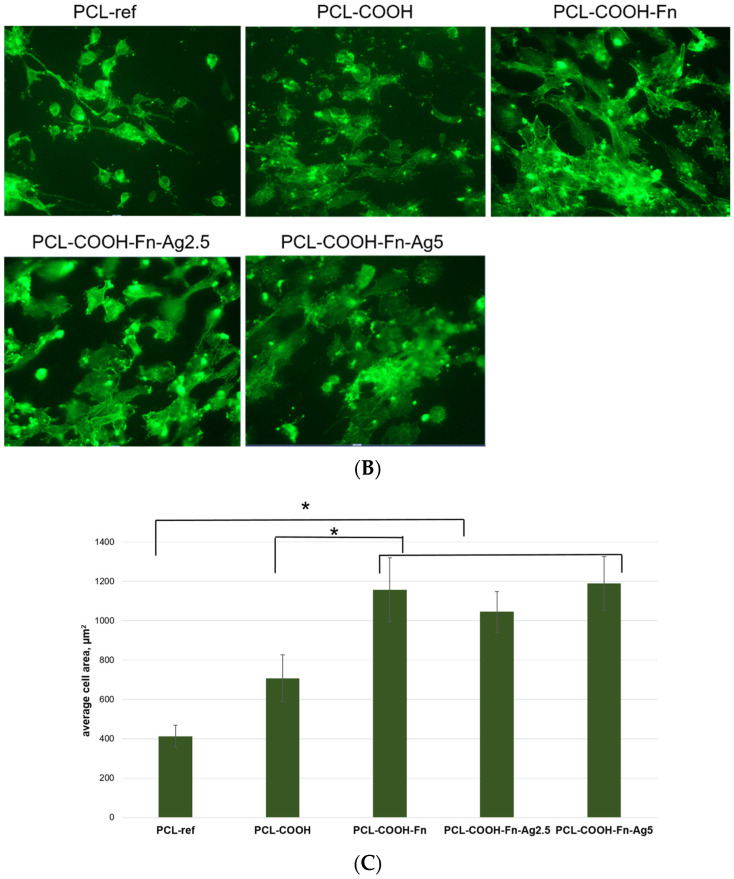
Effect of fibronectin and Ag ion implantation on cell colonization, cell morphology, and formation of mature cytoskeleton. (**A**). The number of cells on the surface after 24 h of cultivation was determined by counting cell nuclei stained with the DNA intercalating dye Hoechst 33342. (**B**). Representative fluorescence photographs of cells cultured on the studied nanofibers. Actin fibers of the cytoskeleton were stained with Phalloidin 488. (**C**). Average areas of fibroblast cells measured by phalloidin staining of cellular actin filaments. * *p* ≤ 0.1.

We have determined the number of cells on the surface of nanofibers after 24 h of cultivation. The results (Figure 6) show that the number of cells is significantly lower on the surface of the PCL-ref sample compared to the PCL—COOH, PCL—COOH-Fn, PCL—CO OH—Fn-Ag2.5, and PCL-COOH-Fn-Ag5 counterparts. These results are comparable with our previous data and are explained by the increased surface hydrophilicity (in the case of PCL-COOH), as well as the additional effect of fibronectin, an active component of the ECM necessary for effective cell adhesion. Surface bombardment with Ag ions, described in previous sections, shows a decrease in the concentration of fibronectin. However, the number of attached, proliferating, viable cells on the PCL-COOH-Fn-Ag2.5 and PCL-COOH-Fn-Ag5 surfaces is not significantly different from the PCL-COOH-Fn material.

Figure 6C demonstrates the significant effect of surface composition on cell spreading area. Thus, the minimum cell size is observed on the untreated hydrophobic surface of the PCL-ref material (413 ± 55 μm^2^), while modification with COOH groups significantly increases the adhesiveness and cell spread area (706 ± 119 μm^2^). These results are in good agreement with our previous data [13]. Surface coating with fibronectin does not affect the total number of cells but significantly increases the cell area (1156 ± 163 μm^2^) and also improves the organization of the actin cytoskeleton, which indicates high fibroblast activity (Figure 6B,C). There is no significant difference in the cell area on the surface of the PCL-COOH-Fn, PCL—COOH—Fn-Ag2.5, and PCL-COOH-Fn-Ag5 samples; however, the percentage of cells with a developed cytoskeleton is lower in the PCL-COOH-Fn-Ag2.5 material (Figure 6B).

Thus, the obtained results show that the fibronectin layer improves the cell spreading and the organization of their actin cytoskeleton, which affects the cell functional activity. Although Ag bombardment significantly reduces the amount of fibronectin on the surface due to its partial degradation, as shown by XPS analysis, the number of cells is not statistically different from unmodified fibronectin. Silver ion implantation into nanofibers can stimulate MSC proliferation and increase cell density [7]. An increase in cell proliferative activity when exposed to Ag ions is associated with stimulation of intracellular ROS generation [31]. It is interesting to note that the PCL-COOH-Fn-Ag2.5 and PCL-COOH-Fn-Ag5 materials exhibit similar adhesion and cell growth dynamics as the PCL-COOH-Fn counterpart. Thus, functionalized materials PCL-COOH-FBn and PCL-COOH-FBn-Ag have good prospects for use in wound healing as they provide good cell adhesion and spreading, stimulate the proliferation of fibroblast cells, and provide antibacterial protection. The combination of all these properties is important in soft tissue regeneration.

### 3.4. Antipathogen Activity

Table 3 presents the summarized data on the antipathogen activity of tested samples against bacterial and fungal strains. It was shown that samples PCL-Fbn-Ag2.5 min, PCL-GM-Ag2.5 min, and PCL-GM had the highest antibacterial activity. It should be noted that Ag-ion implantation during 2.5 min increases the antibacterial activity of samples; however, samples with 5 min Ag-ion implantation demonstrated some negative effects, reducing the size of the inhibition zone. This effect can be explained by the degradation of immobilized molecules, as proven by XPS. In most cases, the tested sample only inhibited the growth of the strain without suppressing it (Figures S1–S13; see supporting information). Meanwhile, the growth of Candida auris KA10, *Klebsiella pneumoniae* 67565/23, and *Proteus mirabilis* 3223/23 strains was neither suppressed nor inhibited by any drug. Thus, although the synergetic effect of silver implantation and gentamycin immobilization was proven, the stimulation of the antipathogen effect for bacteria with resistance or more requires either higher doses of silver ions and drugs or additional functionalities available at the surface.

## 4. Discussion

To create innovative materials for wound healing, it is often necessary to combine several physical and chemical modification methods. Proteins, such as enzymes, growth factors, and ECM components, play crucial roles in biological processes. However, if proteins or therapeutic agent immobilization is combined with physical modification methods such as plasma polymerization or ion implantation, changes in surface composition and structure can be expected. Protein degradation during ion implantation can affect the functionality and bioactivity of wound dressing materials. Here, we show that robust and rapid XPS fitting analysis can be used to control surface structure at each modification step. We fabricated PCL nanofibers coated with a COOH plasma layer, attached fibronectin and gentamycin through chemical bonding, and implanted Ag ions.

CasaXPS software was used to fit the XPS C1s signal of sample PCL-COOH-FBn. The fitted PCL-COOH-FBn spectrum showed that more than 30% of the surface is covered with components that make up fibronectin. The correlation between the nitrogen atomic concentration and the N-C=O and C-N/C-O concentrations confirmed the validity of our fitting method. The N1s signal confirmed that the PCL-COOH-GM surface consists of C-N and NH_3_^+^ environments, i.e., amines and protonated amines. XPS fitting methodology was also used to analyze the surface-modified nanofibers after Ag ion implantation. The results showed significant changes in the composition of PCL-COOH-GM and PCL-COOH-FBn nanofibers, with lower nitrogen concentrations indicating chemical degradation. Analysis of the XPS C1s spectra showed that part of the protein was degraded to form graphite or hydrocarbon. The XPS analysis showed that more than 60% of the protein was degraded, mainly towards hydrocarbons.

It is also worth noting that although the fibronectin molecules were partly degraded, the biological activity of the PCL-COOH-Ag2.5 and PCL-COOH-Ag5 samples was very high. Note that the fibers with a higher Ag content showed slightly higher activity, although the results were not statistically significant. This may be due to the positive effect of Ag ions, which induce ROS generation [21]. Thus, our results confirm that complex synthesis of composite nanofibers combining protein/antibiotic immobilization and Ag ion implantation can stimulate multiple beneficial effects.

One can assume that a sufficiently high Ag content in our nanofibers will provide high antibacterial activity since even at a lower Ag concentration, noticeable antibacterial activity and the absence of biofilm formation were reported [28]. Here, we demonstrated that both silver ions implantation and immobilization of gentamycin may induce significant antibacterial effects against a very broad range of Gram-positive and Gram-negative bacteria. The combination of silver implantation with the immobilization of gentamycin led to a synergetic effect, as the highest antipathogen activity was exhibited by PCL-COOH-GM-Ag2.5 min. However, the increase in the implantation time led to the deterioration of the antibacterial properties. This effect can be related to the degradation of the gentamycin on the one hand and the deep implantation of silver on the other hand. Hence, the optimization of the implantation conditions can be a crucial step for the development of nanofibers with enhanced antibacterial properties. Indeed, our nanofibers exhibited significantly higher antipathogen activity as compared to the numerous recently developed nanofibers with immobilized drugs, such as amikacin [32], oils [33], and other drugs [34].

Although the developed materials exhibited very promising results against a wide range of pathogens, our nanofibers did not show activity against fungal pathogens or bacteria with high resistance. In order to stimulate enhanced effects, it is possible to consider other types of nanofibers, e.g., polyethylene oxide (PEO) of chitosan (CS). Indeed, PEO/CS nanofibers with or without Ag nanoparticles demonstrated antifungal activity [34].

Finally, it should be noted that all processes of nanofiber surface modification can be easily scaled up, as shown elsewhere [21], and even high cost-efficiency can be expected. Our future studies will focus on a detailed analysis of the antibacterial activity of surface-modified nanofibers with a special focus on the strains with high resistance and testing our materials for wound healing in vivo.

## 5. Conclusions

This study demonstrates the efficiency of a new XPS fitting methodology to monitor the surface structure of PCL nanofibers during their sequential modification with a COOH plasma layer, fibronectin/gentamycin, and Ag ion implantation. Immobilization of fibronectin resulted in the formation of peptide bonds. After implantation of Ag ions, which passed through the nanofibers during long-term treatment, significant changes were observed in the topmost surface layer of fibronectin- and gentamicin-immobilized nanofibers containing up to 0.3 at.% of Ag. Estimates have shown that more than 60% of the surface layer has degraded, producing predominantly hydrocarbons. Despite the significant degradation of fibronectin molecules, the biological activity of Ag-implanted nanofibers was high, which is associated with the positive effect of Ag ions, inducing the generation of reactive oxygen species. The PCL nanofibers with immobilized gentamicin and implanted silver ions exhibited very significant antipathogen activity to a wide range of Gram-positive and Gram-negative strains. This process can be easily scaled up to achieve high economic efficiency. Future studies will focus on a detailed analysis of the antibacterial activity of surface-modified nanofibers and in vivo testing on wound healing.

## Figures and Tables

**Table 1:** Sample atomic compositions (at.%) derived from EDXS analysis.

Sample	[C], at.%	[O], at.%	[N], at.%	[Ag], at.%	[Pt], at.%	[K], at.%	[P], at.%
PCL-ref	83.1	16.8	0	0	0.1	0	0
PCL-COOH	81.7	18.2	0	0	0.1	0	0
PCL-COOH-FBN	87.6	11.4	0.5	0	0.1	0.2	0.2
PCL-FbN-Ag-2.5 front side	86.7	12.6	0.2	0.2	0.1	0.1	0.1
PCL-FBN-Ag-2.5 back side	88.5	11.2	0	0	0.1	0.1	0.1
PCL-FBN-Ag-5 front side	86.1	13.2	0.1	0.3	0.1	0.1	0.1
PCL-FBN-Ag-5 back side	85.1	14.5	0	0.1	0.1	0.1	0.1

**Table 2:** Composition of samples (at.%) derived from XPS analysis.

Sample Designation	[C], at.%	[O], at.%	[N], at.%	[S], at.%	[Ag], at.%
PCL-ref	73.9	26.1	0.0	0.0	0.0
PCL-COOH	68.6	30.4	1.0	0.0	0.0
PCL-COOH-FBn	67.1	24.9	8.0	0.0	0.0
PCL-COOH-FBn-Ag2.5	80.8	13.8	5.3	0.0	0.1
PCL-COOH-GM	69.4	27.3	2.9	0.5	0.0
PCL-COOH-GM-Ag2.5	82.6	16.3	1.0	0.2	0.4

**Table 3:** Zones of growth suppression of fungal and bacterial strains by the tested samples. Please note I stands for the inhibition of growth with no measurable inhibition zone.

Strain/Sample	Diameter of Inhibition Zone, mm							
	ref	PCL-COOH	PCL-FBN	PCL-FBN-Ag 2.5 min	PCL-FBN-Ag 5 min	PCL-GM	PCL-GM-Ag 2.5 min	PCL-GM-Ag 5 min
*Escherichia coli* U20	i	i	i	i	i	5	7	i
*Escherichia coli* U4	-	-	i	7	-	5	11	-
*Escherichia coli* ATCC25922	-	-	i	6	i	5	8	i
*Escherichia coli* K261	-	-	i	-	i	i	-	i
*Staphylococcus aureus* BAA1707 (MW2)	-	-	-	5	-	8	14	-
*Staphylococcus aureus* 11	i	i	i	7	i	11	15	i
*Candida auris* KA10	-	-	-	-	-	-	-	-
*Enterococcus faecium* Ya253	-	i	i	i	i	i	i	i
*Enterococcus faecium* i237	-	i	i	i	i	i	i	i
*Staphylococcus aureus* 10708/23	-	-	-	6	-	9	13	-
*Klebsiella pneumoniae* 67565/23	-	-	-	-	-	-	-	-
*Pseudomonas aeruginosa* 3945/23	-	-	-	-	-	-	i	-
*Proteus mirabilis* 3223/23	-	-	-	-	-	-	-	-

## Data Availability

Data is available from the corresponding author upon a reasonable request.

## Supplementary Materials

The following supporting information can be downloaded at: https://www.mdpi.com/article/10.3390/polym16020261/s1. Figure S1. Photo of petri dishes with a “lawn” of *E. coli* strain U20 when exposed to samples ref (r), 1, 2 and 3 (A,B), and 4, 5, 6 and 7 (C,D). Figure S2. Photo of petri dishes with a “lawn” of *E. coli* U4 strain when exposed to samples ref (r), 1, 2 and 3 (A,B), and 4, 5, 6 and 7 (C,D). Figure S3. Photo of petri dishes with a “lawn” of *E. coli* strain ATCC25922 when exposed to samples ref (r), 1, 2 and 3 (A,B), and 4, 5, 6 and 7 (C,D). Figure S4. Photo of petri dishes with a “lawn” of *E. coli* strain K261 when exposed to samples ref (r), 1, 2 and 3 (A,B), and 4, 5, 6 and 7 (C,D). Figure S5. Photo of petri dishes with a “lawn” of *S. aureus* strain BAA1707 (MW2) when exposed to samples ref (r), 1, 2 and 3 (A,B), and 4, 5, 6 and 7 (C,D). Figure S6. Photo of petri dishes with a “lawn” of *S. aureus* strain 11 when exposed to samples ref (r), 1, 2 and 3 (A,B), and 4, 5, 6 and 7 (C,D). Figure S7. Photo of petri dishes with a “lawn” of *C. auris* strain KA10 when exposed to samples ref (r), 1, 2 and 3 (A,B), and 4, 5, 6 and 7 (C,D). Figure S8. Photo of petri dishes with a “lawn” of E. faecium i237 strain when exposed to samples ref (r), 1, 2 and 3 (A,B), and 4, 5, 6 and 7 (C,D). Figure S9. Photo of petri dishes with a “lawn” of E. faecium strain Ya253 when exposed to samples ref (r), 1, 2 and 3 (A,B), and 4, 5, 6 and 7 (C,D). Figure S10. Photo of petri dishes with “lawn” of *S. aureus* strain 10708/23 when exposed to ref (r), 1, 2, 3, 4, 5, 6 and 7 samples. Figure S11. Photo of petri dishes with a “lawn” of Klebsiella pneumoniae strain 67565/23 when exposed to samples ref (r), 1, 2, 3, 4, 5, 6 and 7. Figure S12. Photo of petri dishes with a “lawn” of Pseudomonas aeruginosa strain 3945/23 when exposed to samples ref (r), 1, 2, 3, 4, 5, 6 and 7. Figure S13. Photo of petri dishes with “lawn” of Proteus mirabilis strain 3223/23 when exposed to samples ref (r), 1, 2, 3, 4, 5, 6 and 7.
