# Supplementary material for: Polycaprolactone Nanofibers Functionalized by Fibronectin/Gentamicin and Implanted Silver for Enhanced Antibacterial Properties, Cell Adhesion, and Proliferation
Source: Polymers (Basel). 2024 Jan 17;16(2):261. doi: 10.3390/polym16020261 (PMC10819432; doi:10.3390/polym16020261)
Supplement: Supplementary file 1 [file polymers-16-00261-s001.zip › polymers-2759092-supplementary.pdf]

polymers

Article

Polycaprolactone Nanofibers Functionalized by Fibronectin/ Gentamicin and Implanted Silver for Enhanced Antibacterial Properties, Cell Adhesion, and Proliferation

Elizaveta S. Permyakova [1], Anastasiya O. Solovieva [2], Natalia Sitnikova [2], Philipp V. Kiryukhantsev-Korneev [1], Magzhan K. Kutzhanov [1], Alexander N. Sheveyko [1], Sergey G. Ignatov [1,3,4], Pavel V. Slukin [1,3], Dmitry V. Shtansky [1] and Anton M. Manakhov [1,2,*]

[1] Research Laboratory "Inorganic Nanomaterials", National University of Science and Technology "MISIS", Moscow 119049, Russia;
permyakova.elizaveta@gmail.com (E.S.P.); kiruhancev-korneev@yandex.ru (P.V.K.-K.); makonyo95year@gmail.com (M.K.K.); sheveyko@mail.ru (A.N.S.); ignatov.sergei@gmail.com (S.G.I.); xopgi@yandex.ru (P.V.S.); shtansky@shs.misis.ru (D.V.S.)
[2] Research Institute of Clinical and Experimental Lymphology—Branch of the ICG SB RAS, 2 Timakova st., Novosibirsk 630060, Russia; solovey_ao@mail.ru (A.O.S.); sitnikovanat9@gmail.com (N.S.)
[3] State Research Center for Applied Microbiology and Biotechnology, Obolensk 142279, Russia
[4] Lomonosov Moscow State University, GSP-1, 1 Leninskiye Gory, Moscow 119991, Russia
* Correspondence: ant-manahov@ya.ru (A.M.)

The samples used in the study were: PCL-ref (r), PCL-Fbn-Ag 5min (1), PCL-Fbn-Ag 2.5min (2), PCL-GM-Ag 2.5min (3), PCL-GM-Ag 5min (4), PCL-COOH (5), PCL-GM (6), PCL-Fbn (7). The Figures S1-S13 demonstrated optical images of samples in Petri dishes after exposure to the pathogens.

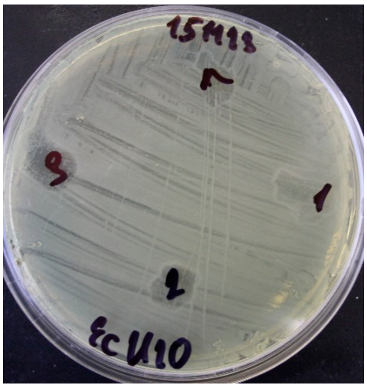	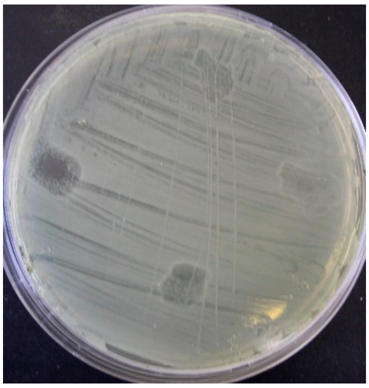
A	B
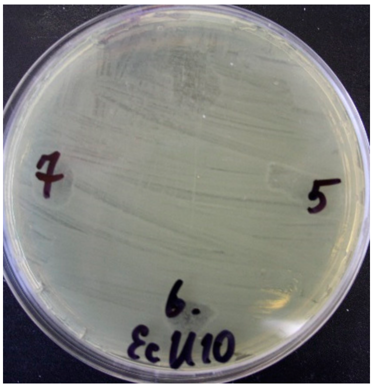	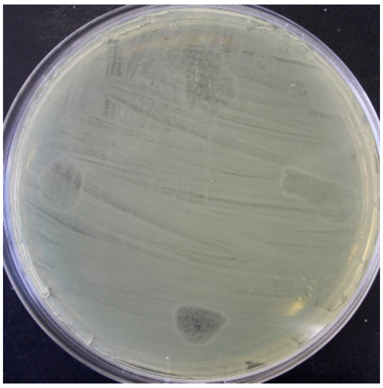
C	D

Figure S1. Photo of petri dishes with *E. coli* strain U20 when exposed to samples ref (r), 1, 2 and 3 (A,B), and 4, 5, 6 and 7 (C,D).

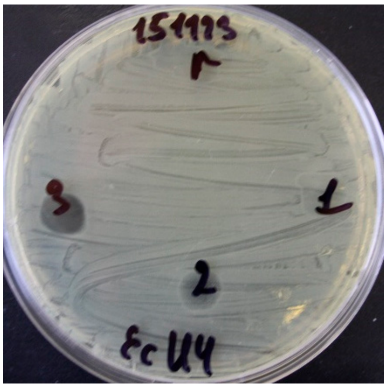	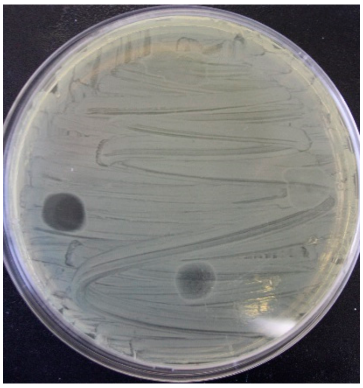
A	B
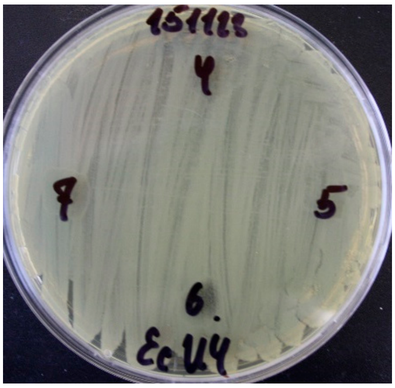	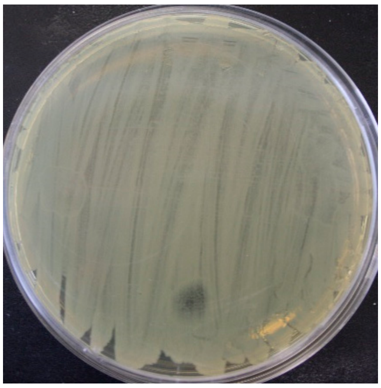

C	D

Figure S2. Photo of petri dishes with *E. coli* U4 strain when exposed to samples ref (r), 1, 2 and 3 (A,B), and 4, 5, 6 and 7 (C,D).

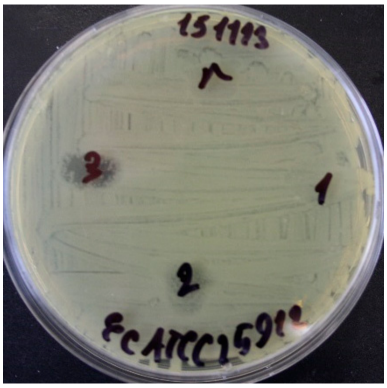	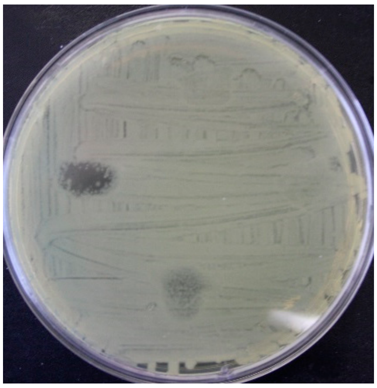
A	B
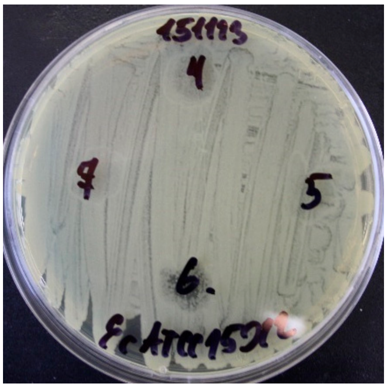	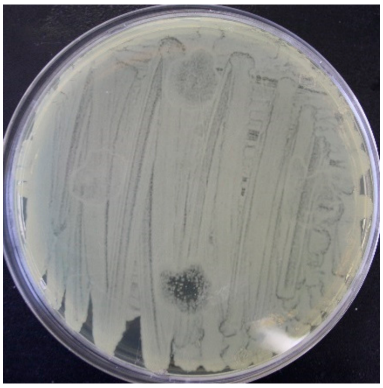
C	D

Figure S3. Photo of petri dishes with *E. coli* strain ATCC25922 when exposed to samples ref (r), 1, 2 and 3 (A,B), and 4, 5, 6 and 7 (C,D).

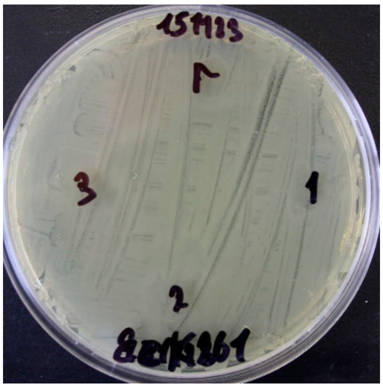	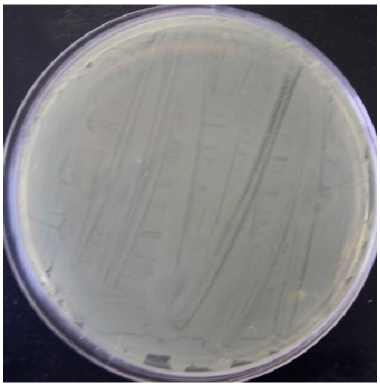
A	B

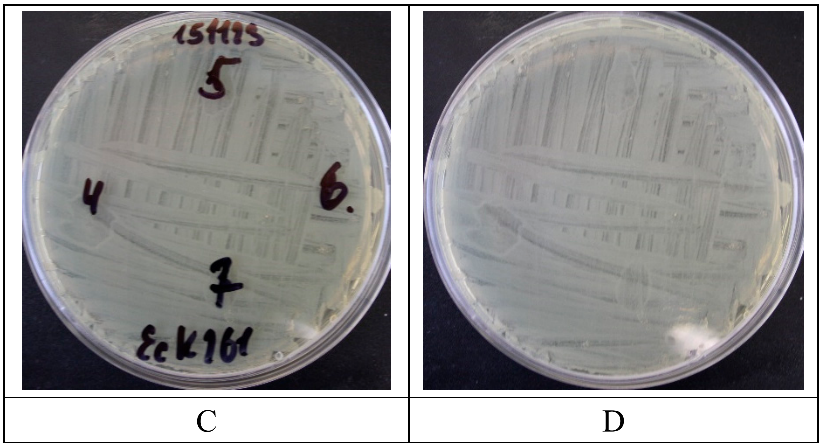

Figure S4. Photo of petri dishes with *E. coli* strain K261 when exposed to samples ref (r), 1, 2 and 3 (A,B), and 4, 5, 6 and 7 (C,D).

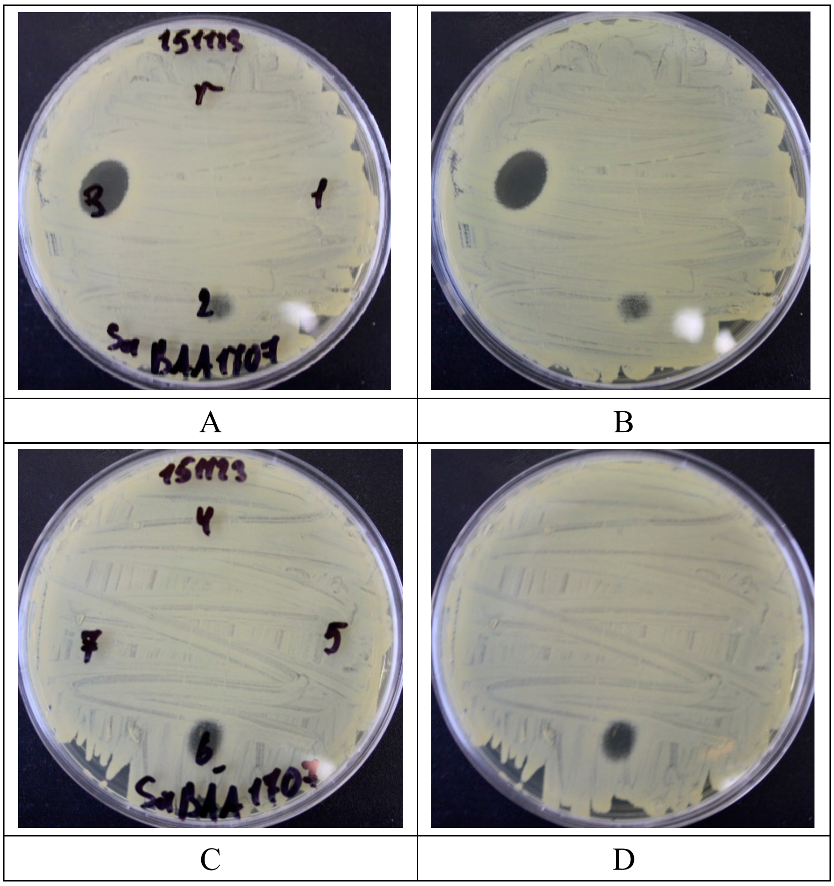

Figure S5. Photo of petri dishes with *S. aureus* strain BAA1707 (MW2) when exposed to samples ref (r), 1, 2 and 3 (A,B), and 4, 5, 6 and 7 (C,D).

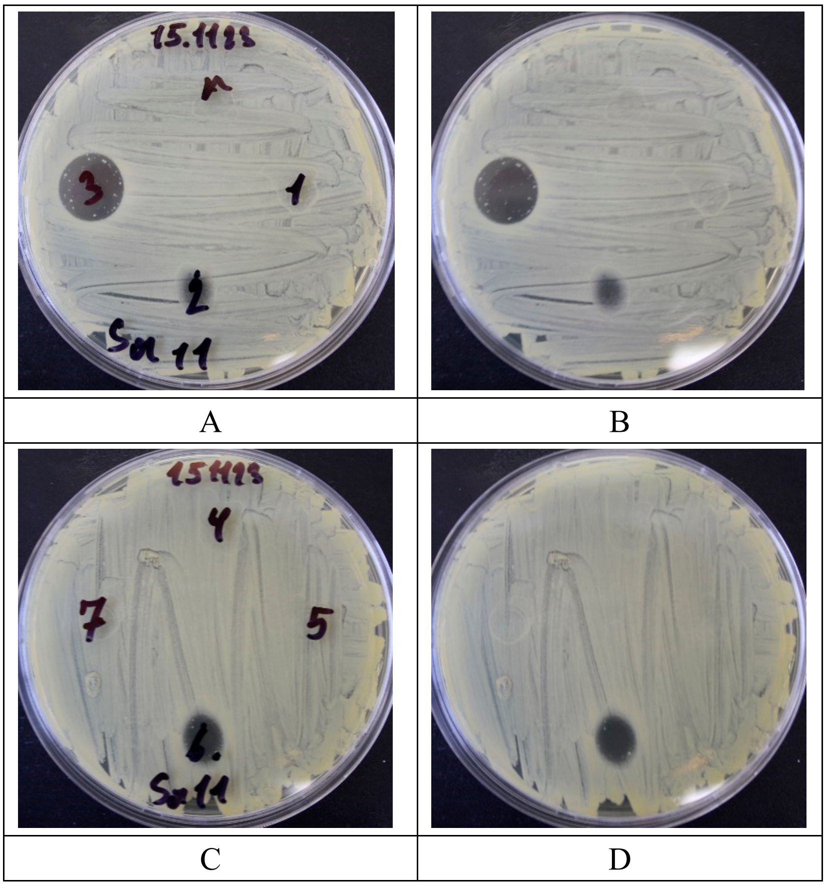

Figure S6. Photo of petri dishes with *S. aureus* strain 11 when exposed to samples ref (r), 1, 2 and 3 (A,B), and 4, 5, 6 and 7 (C,D).

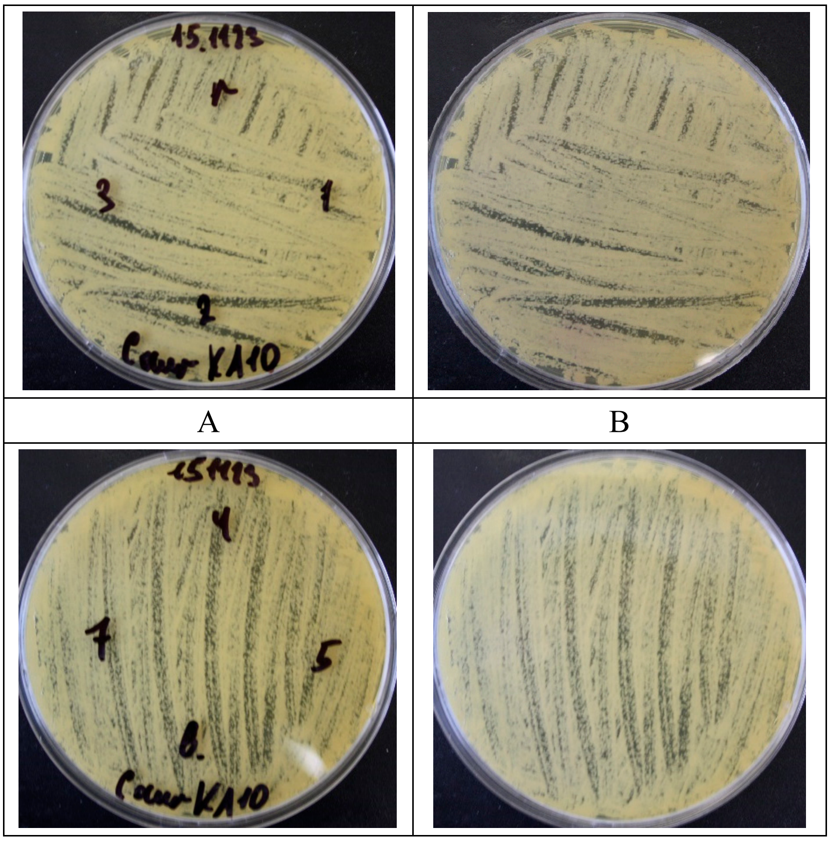

C	D

Figure S7. Photo of petri dishes with *C. auris* strain KA10 when exposed to samples ref (r), 1, 2 and 3 (A,B), and 4, 5, 6 and 7 (C,D).

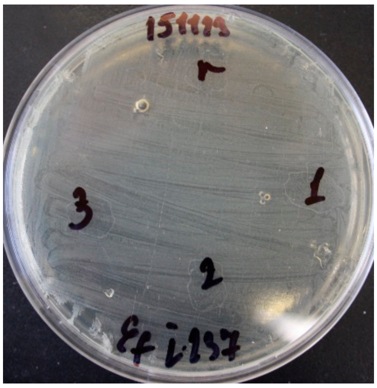	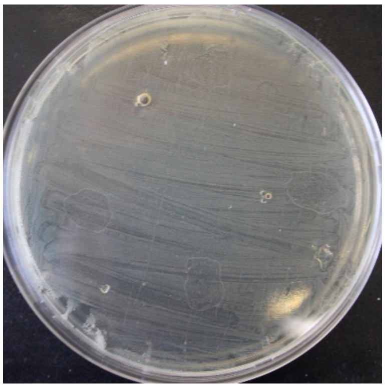
A	B
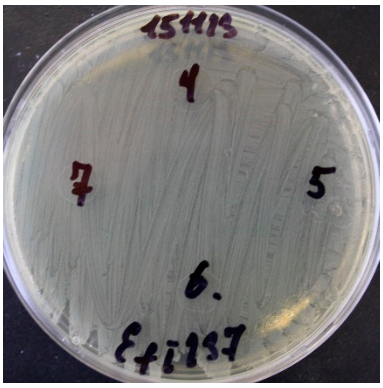	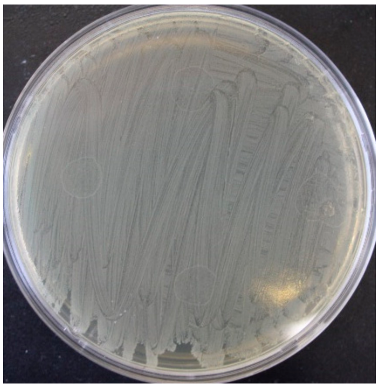
C	D

Figure S8. Photo of petri dishes with *E. faecium* i237 strain when exposed to samples ref (r), 1, 2 and 3 (A,B), and 4, 5, 6 and 7 (C,D).

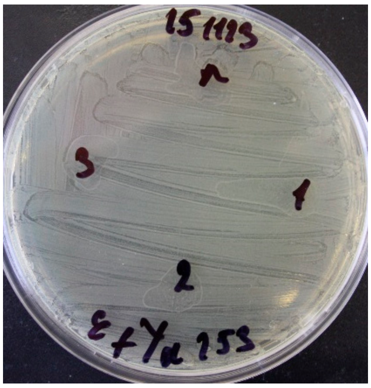	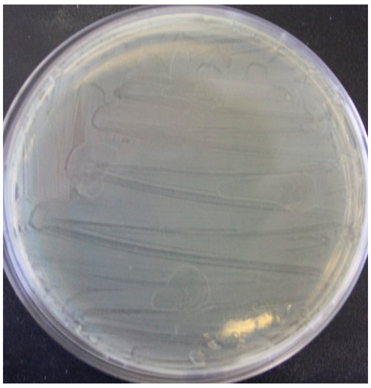
A	B

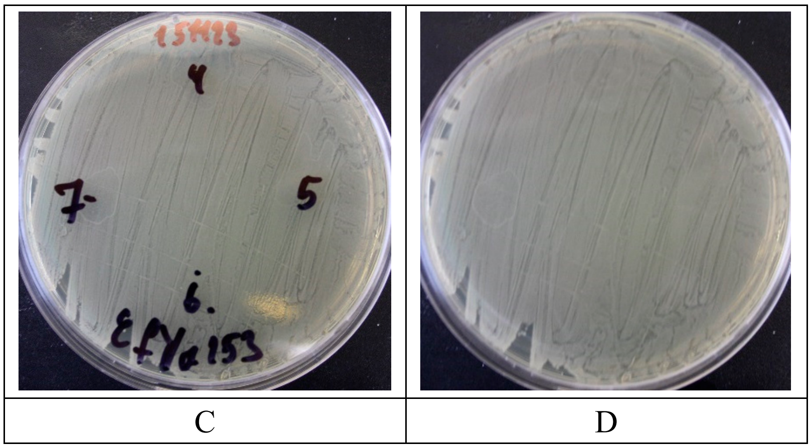

C	D

Figure S9. Photo of petri dishes with *E. faecium* strain Ya253 when exposed to samples ref (r), 1, 2 and 3 (A,B), and 4, 5, 6 and 7 (C,D).

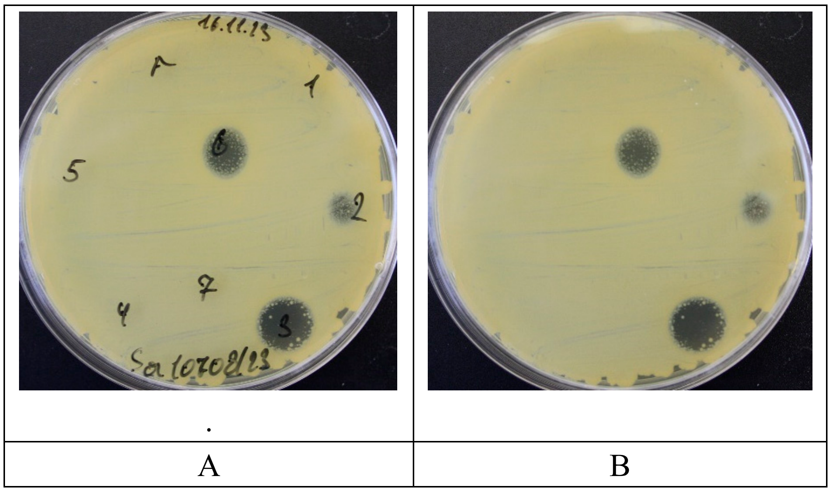

A	B

Figure S10. Photo of petri dishes with *S. aureus* strain 10708/23 when exposed to ref (r), 1, 2, 3, 4, 5, 6 and 7 samples.

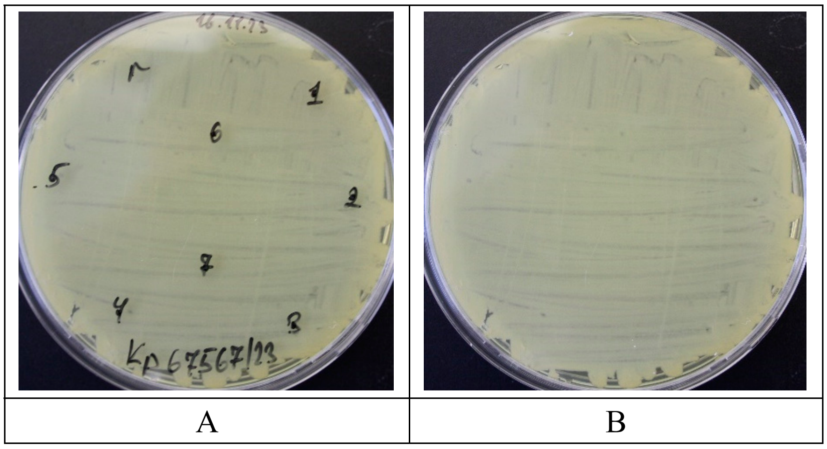

A	B

Figure S11. Photo of petri dishes with Klebsiella pneumoniae strain 67565/23 when exposed to samples ref (r), 1, 2, 3, 4, 5, 6 and 7.

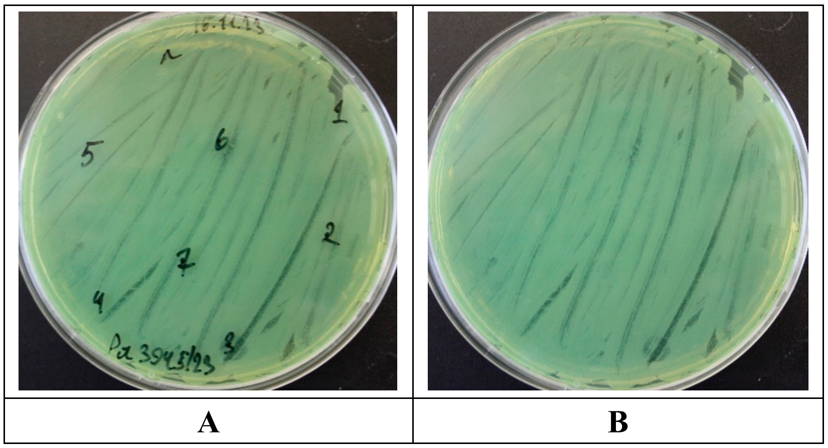

A	B

Figure S12. Photo of petri dishes with Pseudomonas aeruginosa strain 3945/23 when exposed to samples ref (r), 1, 2, 3, 4, 5, 6 and 7.

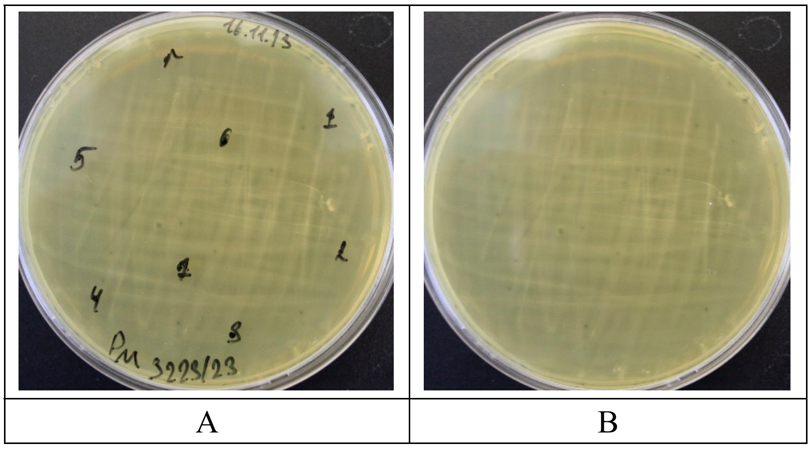

A	B

Figure S13. Photo of petri dishes with Proteus mirabilis strain 3223/23 when exposed to samples ref (r), 1, 2, 3, 4, 5, 6 and 7.

Photo of the same Petri dish with numbers of samples (A- left part) and without numbers (B –right part)
